# Lysine Methyltransferase SETD7 (SET7/9) Regulates ROS Signaling through mitochondria and NFE2L2/ARE pathway

**DOI:** 10.1038/srep14368

**Published:** 2015-10-05

**Authors:** Shuying He, Dafydd R. Owen, Scott A. Jelinsky, Lih-Ling Lin

**Affiliations:** 1 Inflammation and Immunology Research Unit, Pfizer Research, Cambridge, MA 02139; 2 Worldwide Medicinal Chemistry, Pfizer Worldwide Research and Development, Cambridge, MA 02139.

## Abstract

Reactive oxygen species (ROS) homeostasis requires stringent regulation. ROS imbalance, especially ROS accumulation, has profound implications in various disease pathogenesis. Lysine methylation of histone and non-histone proteins has been implicated in various cellular responses. The main objective of this study is to investigate the role of SET domain containing lysine methyltransferase SETD7 (SET7/9) in the regulation of ROS-mediated signaling. Here we report that inhibition of SETD7 with siRNA or a SETD7 small molecule inhibitor in both macrophages and a human bronchial epithelial cell line (Beas-2B) were able to counter NF-ĸB-induced oxidative stress and pro-inflammatory cytokine production. Meanwhile, inhibition of SETD7 elevates mitochondria antioxidant functions *via* negative regulation of PPARGC1A and NFE2L2. Using a co-expression system and purified proteins, we detected direct interaction between SETD7 and NFE2L2. These results indicate that lysine methylation by SETD7 is important for the fine-tuning of ROS signaling through its regulation on pro-inflammatory responses, mitochondrial function and the NFE2L2/ARE pathway. Up-regulation of multiple antioxidant genes and improved ROS clearance by inhibition of SETD7 suggests the potential benefit of targeting SETD7 in treating ROS-associated diseases.

Lysine methylation is critical for the regulation of both transcription and protein functions. Methylation of different lysine residues at histone tails can serve either as an activating or repressive code to mediate topological changes in individual nucleosomes and direct chromatin dynamics^1^,^2^. SET domain containing lysine methyltransferase 7 (SETD7, also called SET7/9) was the first lysine methyltransferase (KMT) discovered to specifically monomethylate lysine-4 of histone 3 (H3K4me1), a marker for transcriptional activation^2^,^3^. Interestingly, SETD7 can also methylate a number of non-histone proteins such as p53, TAF10, ER, P65, STAT3, SOX2, pRb, SIRT1, DNMT1, SUV39H1 and FOXO3^4^,^5^,^6^,^7^,^8^,^9^,^10^,^11^,^12^,^13^,^14^. To date, how SETD7 coordinates its functions in transcriptional activation and its regulatory effects on non-histone substrates remains unclear. SETD7 has been implicated to be involved in various signaling or disease pathways^15^,^16^,^17^. Surprisingly, SETD7 knockout mice are phenotypically normal and they do not carry apparent deficiencies in DNA damage and oncogene-induced p53 responses^18^,^19^. These findings indicate that instead of direct control of physiological functionalities, SETD7 may participate in sensing and adjusting signaling events in response to the dynamic changes within the cellular contexts.

Reactive oxygen species (ROS) have dual roles in living organisms. While a low concentration of ROS can act as important signaling molecule, accumulation of ROS is a threat to cellular activities^20^. Endogenous ROS can originate from metabolic processes such as glycolysis, gluconeogenesis, lipid metabolism and ATP or nitric oxide synthesis. ROS neutralization primarily depends on antioxidant defense through a variety of ROS detoxifying enzymes. Imbalance between the redox molecules and antioxidants can trigger or exacerbate cytotoxic effects, which ultimately leads to various diseases including aging, metabolic dysfunctions, neurodegeneration, chronic inflammation, cardiovascular defects and oncogenesis^20^,^21^,^22^. Mitochondrial-derived ROS accounts for the majority of total ROS within cells. Mitochondrial ROS neutralization mainly depends on two mitochondrial ROS scavenger enzymes: manganese-containing superoxide dismutase (MNSOD or SOD2) and catalase (CAT)^23^. In addition, the metabolic regulator peroxisome proliferator activated receptor gamma, coactivator 1 Alpha (PPARGC1A or PGC-1α), which orchestrates a series of mitochondrial activities including mitochondria biogenesis and antioxidant responses, is indispensable for mitochondrial functional integrity^24^,^25^,^26^,^27^,^28^,^29^. Nuclear factor erythroid 2-like 2 (NFE2L2 or NRF2) Antioxidant Responsive Elements (ARE) pathway is considered as the cornerstone of the antioxidant defense system^30^,^31^,^32^,^33^,^34^,^35^. The majority of antioxidant genes including *heme oxygenase 1* (*HMOX1*), *thioredoxin* (*TXN*), and *peroxiredoxin-I* (*PRDXI*) contain AREs at or proximal to their promoters^30^,^31^,^36^,^37^. Under ROS stimulation, NFE2L2 is released from its cytosolic repressor, Kelch-like ECH-associated protein 1 (KEAP1) and E3 ubiquitin ligase cullin 3 (CUL3) facilitating its translocation into the nucleus, where it binds to AREs at the promoters of its target genes to enable their expression^32^,^33^,^38^. Besides ubiquitination, NFE2L2 is also a substrate for phosphorylation and acetylation^39^,^40^,^41^. Nevertheless, post translational modifications that regulate the activity of NFE2L2 are not fully described. Characterization of the post-translational modifications on the NFE2L2 protein will be important for understanding the regulatory mechanisms within the NFE2L2/ARE pathway.

In this study we discovered novel roles of SETD7 lysine methyltransferase in the regulation of ROS signaling via regulation of NF-ĸB and proinflammatory cytokine production, PPARGC1A, and NFE2L2 expression. Our results suggest that SETD7 is important in regulating ROS signaling and mitochondria.

## Results

### Oxidative stress-induced NF-ĸB activity is attenuated by inhibition of SETD7

**Figure 1:**
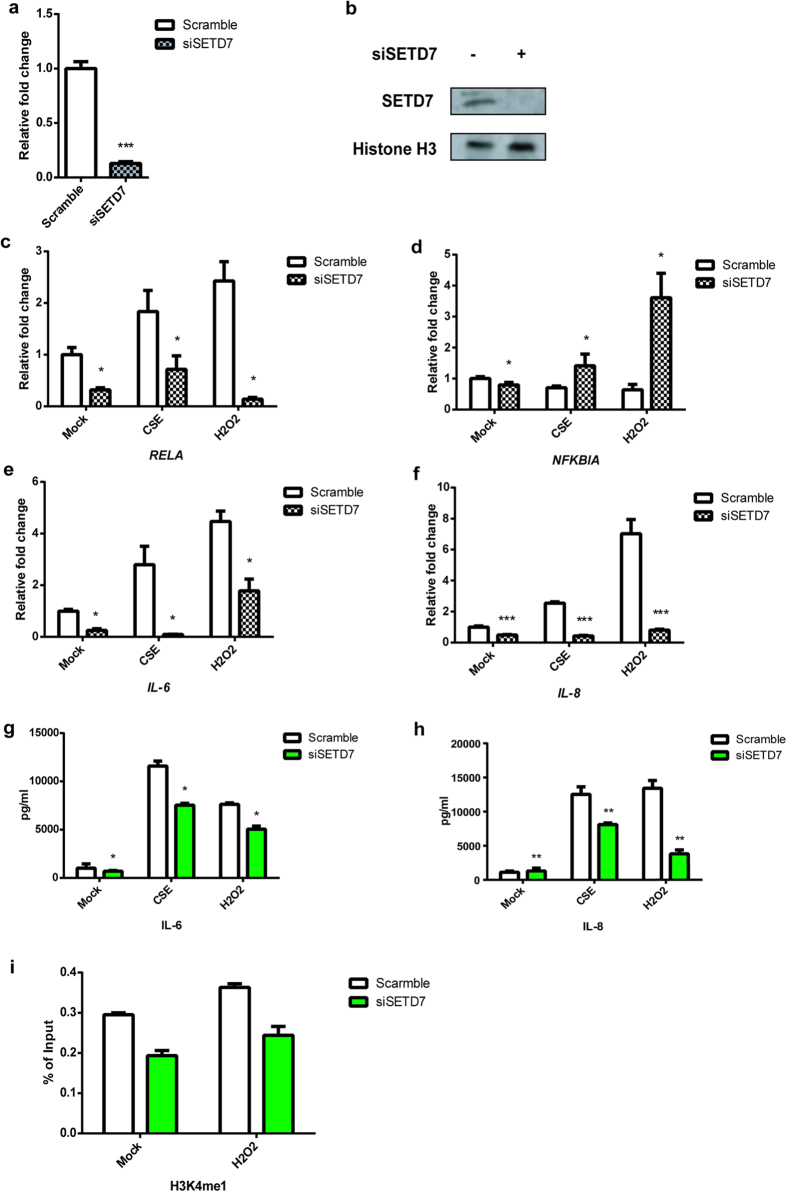
Knockdown of SETD7 represses NF-ĸB and the expression of proinflammatory cytokine under oxidative stress. siRNA knockdown of SETD7 in Beas-2B cells was assessed by qRT-PCR (**a**) and western blot using antibody against SETD7 (**b**). *** indicates *P* < 0.001 (Student’s unpaired t test). (**c–f**) Transcriptional level of *RELA*, *NFKBIA*, *IL-6* and *IL-8* was measured by qRT-PCR after SETD7 siRNA transfection under unstimulated, CSE- or H_2_O_2_-stimulated conditions. Relative fold change of mRNA level was quantified by comparison with cells transfected with scramble siRNA without stimulation (mock). (**g,h**) IL-6 and IL-8 cytokine production under normal and ROS-stimulated condition was quantified by MSD. (**i**) Relative enrichment of H3K4me1 at the promoter region of *RELA* by qChIP. Beas-2B cells treated with scramble or siSETD7 siRNA were stimulated by H_2_O_2_ and their chromatin was fixed for the experiments. The level of H3K4me1 was shown by % of input of each individual sample. Data were expressed as mean ± standard error of the mean (SEM). Relative fold change was calculated by comparing each sample group with scramble siRNA-treated cells without stimulation. Two negative control sites locate at the gene desert were included for each experiment. n = 3. * indicates *P* < 0.05; ** indicates *P* < 0.01 (two-way ANOVA).

SETD7 has been found to regulate *RELA* mainly through H3K4me1 in several studies^7^,^15^. To characterize the roles of SETD7 in NF-ĸB-dependent oxidative stress, we performed siRNA knockdown in primary human GM-CSF derived macrophages and in human bronchial epithelial cell line Beas-2B followed by cigarette smoke extract (CSE) or hydrogen peroxide (H_2_O_2_) stimulation. Knockdown efficiency was determined by both qPCR and western blot (Fig. 1a,b). Consistent with other studies^18^,^42^, SETD7 silencing did not seem to affect total H3K4me1 levels (see Supplementary Fig. S1 online). In both Beas-2B and macrophages, both CSE and H_2_O_2_ caused up-regulation of *RELA* and inhibition of *NFKBIA*, while knockdown of SETD7 was able to repress basal and CSE- and H_2_O_2_- induced expression of *RELA* as well as pro-inflammatory cytokines *IL-6* and *IL-8* (Fig. 1c–h; see Supplementary Fig. S1 online). Meanwhile, chromatin immunoprecipitation (ChIP) was performed to determine if SETD7 affects the transcriptional activity of *RELA* through H3K4me1. Treatment of Beas-2B cells with H_2_O_2_ increase H3K4me1 levels at the *RELA* promoter which was decreased by inhibition of SETD7 (Fig. 1i). These results indicate that activation of NF-ĸB by CSE or H_2_O_2_-induced oxidative stress is SETD7-dependent. Under oxidative stress, SETD7 is able to regulate the transcription activity of *RELA* through H3K4me1.

### SETD7 silencing enhances ROS clearance and upregulates enzymes involved in mitochondria protection

**Figure 2:**
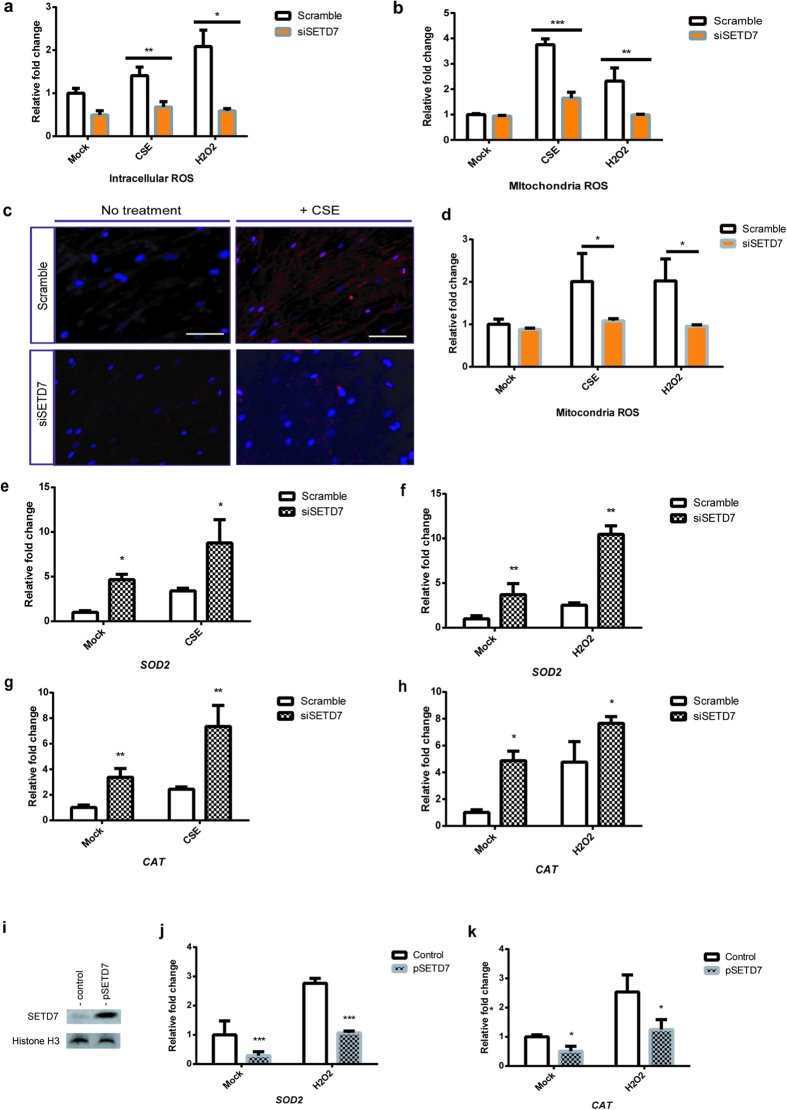
SETD7 silencing facilitates ROS clearance. (**a**) Intracellular ROS in both scramble and SETD7 knockdown Beas-2B cells were measured by cell-permeable fluorogenic probe DCFH-DA. (**b**) Mitochondrial superoxide level was quantified by MitoSox in Beas-2B cell in the presence of SETD7 siRNA. (**c,d**) Mitochondrial superoxide accumulation was visualized and quantified in NHLFs using MitoSox live cell imaging dye (red). Nuclei were counterstained with DAPI (blue). Scale bar = 50 μM. * indicates *P* < 0.05. (**e–h**) Transcriptional levels of *superoxide dismutase 2* (*SOD2)* and *catalase* (*CAT*) was determined by qRT-PCR in the SETD7 knockdown Beas-2B cells under CSE- or H_2_O_2_ -stimulated condition. (**i**) Overexpression of SETD7 in Beas-2B cells after transfection of SETD7 wild type plasmid was determined by Western blot. (**j,k**) *SOD2* and *CAT* mRNAs were quantified by qRT-PCR after transfection of pSETD7 vectors and oxidative stress induction by H_2_O_2_. Data were expressed as mean ± standard error of the mean (SEM). n = 3. * indicates *P* < 0.05; ** indicates *P* < 0.01; *** indicates *P* < 0.001 (two-way ANOVA).

Since smoke-induced ROS accumulation may be the major contributor to the pathology associated with Chronic Obstructive Pulmonary Disease (COPD)^21^,^43^, we examined whether inhibition of SETD7 can functionally improve the cellular detoxification capacity in Beas-2B cells and primary human lung fibroblasts (NHLFs). Cellular ROS levels were measured by cell-permeable fluorogenic probe 2′, 7′-Dichlorodihydrofluorescin diacetate (DCFH-DA). Both CSE and H_2_O_2_ stimulated a burst of intracellular and mitochondrial ROS in Beas-2B cells whereas knockdown of SETD7 attenuated both basal and induced ROS (Fig. 2a,b). Furthermore, mitochondrial superoxide in live NHLFs was increased following stimulation with CSE and this inhibition was blocked by knockdown of SETD7 (Fig. 2c,d). Decreased superoxide production may result from increased expression of superoxide dismutase 2 (SOD2) and catalase (CAT). In Beas-2B cells, we observed an upregulation on the transcription of *SOD2* and *CAT* after CSE or H_2_O_2_ stimulation. Interestingly, the transcriptional levels of these two mitochondrial ROS scavenger enzymes were further increased in the SETD7 knockdown cells (Fig. 2e–h). To confirm that transcriptional activity of *SOD2* and *CAT* can be regulated by SETD7, we overexpressed SETD7 in Beas-2B cells (Fig. 2i). Our data demonstrated that overexpression of SETD7 can attenuate the transcription activity of *SOD2* and *CAT* (Fig. 2j,k).

### SETD7 silencing improves overall mitochondrial functions through PPARGC1A

**Figure 3:**
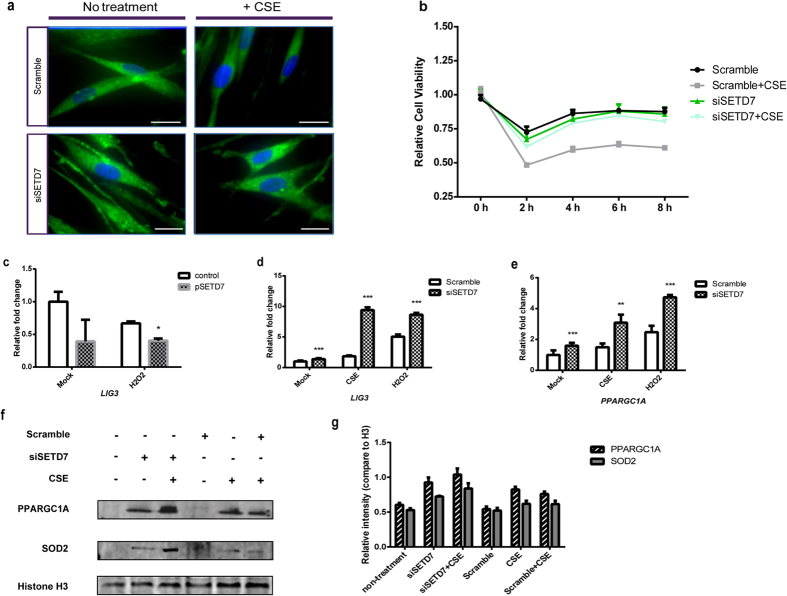
SETD7 regulates mitochondria stability through PPARGC1A. (**a**) Mitochondria population was visualized by MitoTracker (green) in NHLFs with or without CSE stimulation. Nuclei were counterstained with DAPI (blue). Scale bar = 100 μm. (**b**) Cell viability of NHLFs over time course with or without CSE-induced oxidative stress was assessed by WST-1. The tetrazolium salt WST-1 can be cleaved by mitochondrial dehydrogenases in live cells and form formazan dye in proportion to the number of viable cells. (**c,d**) *DNA Ligase III* (*LIG3*) mRNA level was quantified by qRT-PCR in Beas-2B cells with either ectopic expression of SETD7 or siSETD7 knockdown. CSE or H_2_O_2_ was added into the media to induce oxidative stress. (**e**) *PPARGC1A* mRNA in SETD7 knockdown Beas-2B cells treated by either CSE or H_2_O_2_ was measured by qPCR. (**f,g**) Western blot was performed to quantify PPARGC1A and SOD2 protein expression in the SETD7 knockdown Beas-2B cells under CSE-induced oxidative stress. Relative expression of both proteins was compared with Histone H3 control in each individual experiment. Data were expressed as mean ± standard error of the mean (SEM). n = 3. * indicates *P* < 0.05; ** indicates *P* < 0.01 (two-way ANOVA).

Reduction of ROS in the SETD7 knockdown cells prompted us to ask how SETD7 regulates mitochondrial ROS. Using mitoTracker as a mitochondria marker, we observed that siSETD7 knockdown cells displayed slightly higher mitochondria signal when compared with control cells transfected with scramble siRNAs (Fig. 3a). Furthermore, CSE-induced oxidative stress led to mitochondrial fragmentation as reflected by the attenuation of mitoTracker signal. However, inhibition of SETD7 by siRNA was able to protect mitochondria against CSE-induced mitochondria pathology (Fig. 3a). In addition, SETD7 silencing was able to improve overall cell survival against CSE-induced cytotoxicity (Fig. 3b). DNA Ligase III (LIG3) is critical to mitochondria genomic stability and cell viability^44^,^45^. Under oxidative stress induced by CSE or H_2_O_2_, *LIG3* mRNA levels were increased in the SETD7 knockdown Beas-2B cells but were repressed when SETD7 was overexpressed (Fig. 3c,d). Interestingly, up-regulation of *PPARGC1A,* a regulator of mitochondrial biogenesis and functions, was observed in the SETD7 knockdown Beas-2B cells even without oxidative stress challenges (Fig. 3e). Up-regulation of both SOD2 and PPARGC1A protein expression was found in the SETD7 knockdown Beas-2B cells, suggesting that SETD7 inhibition can promote the expression mitochondrial ROS scavenger enzymes through up-regulation of PPARGC1A (Fig. 3f,g). These observations indicate that SETD7 is involved in the regulation of mitochondrial functions by affecting the expression of PPARGC1A.

### SETD7 negatively regulates NFE2L2 and interacts with NFE2L2

**Figure 4:**
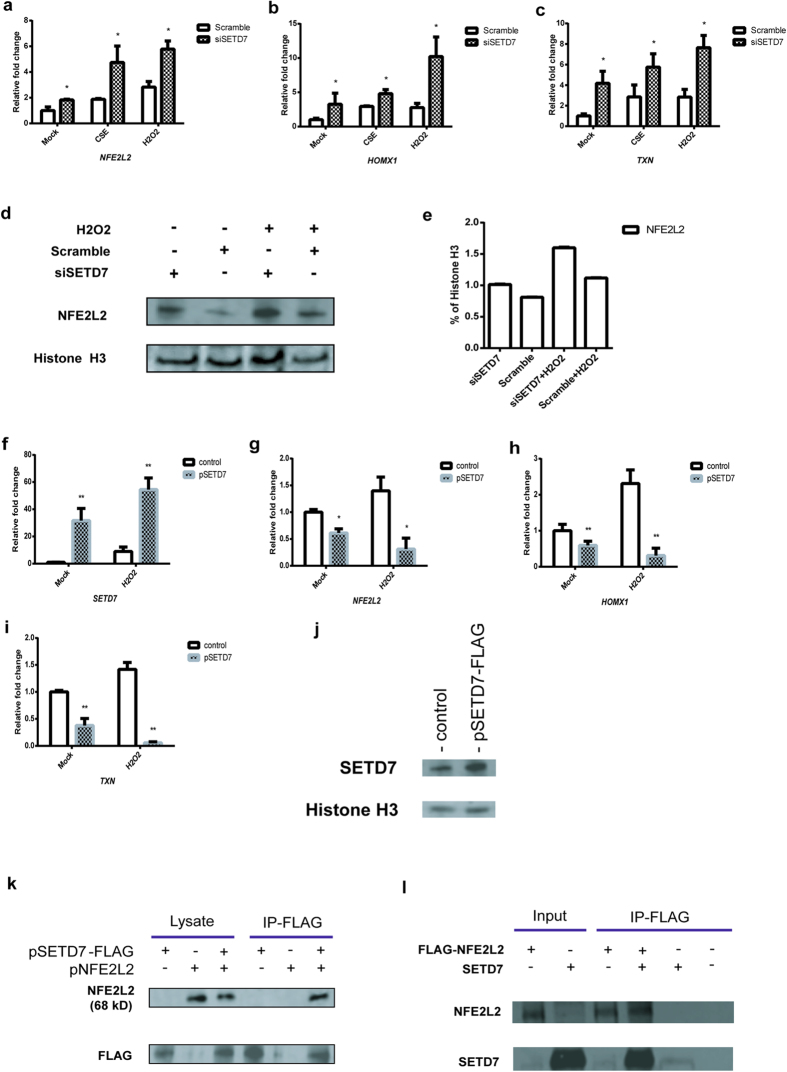
SETD7 negatively regulates NFE2L2/ARE pathway. (**a–c**) Total RNA was isolated from SETD7 knockdown Beas-2B cells to determine the transcription levels of *NFE2L2*, heme oxygenase 1 (*HMOX1*) and thioredoxin (*TXN*). CSE or H_2_O_2_ was added to induce oxidative stress. (**d,e**) NFE2L2 protein in SETD7 knockdown Beas-2B cells was quantified by western blot. H_2_O_2_ was used to induce ROS. Relative expression level of NEF2L2 was measured by comparing with the expression of Histone H3 control in each individual experiment. (**f–i**) Transcription of *SETD7, NFE2L2, HMOX1* and *TXN* was quantified by qRT-PCR using total RNAs isolated from Beas-2B cells with overexpression of SETD7 and H_2_O_2_ stimulation. (**j**) Expression of FLAG-tagged SETD7 plasmid was confirmed by western blot using HEK293 cell lysate after 48 hs transfection. (**k**) Protein pull down assay was performed using lysates from HEK293 cells cotransfected with FLAG-tagged SETD7 and untagged NFE2L2 wild type vectors. Transfection of FLAG-tagged SETD7 vectors or empty FLAG-tagged vectors (control) was included in the experiments. Detection of NFE2L2 protein was performed with western blot using anti-NFE2L2 primary antibody. (**l**) *in vitro* protein pull-down assay was performed using FLAG-tagged human recombinant NFE2L2 protein and GST-tagged human recombinant SETD7 protein. Detection of both proteins was visualized by western blot using anti-SETD7 and anti-NFE2L2 antibodies. Data were expressed as mean ± standard error of the mean (SEM). n = 3. * indicates *P* < 0.05; ** indicates *P* < 0.01 (two-way ANOVA).

By analyzing expression of genes in sputum from ex-smoker COPD patients, we observed an inverse correlation on the transcriptional levels of *SETD7* and *NFE2L2* (see Supplementary Fig. S2 online). This is consistent with our observation that oxidative stress induced by either CSE or H_2_O_2_ stimulated NFE2L2 as well as its downstream targets heme oxidase 1 (HMOX1) and thioredoxin (TXN). Inhibition of SETD7 was able to further increase the expression of NFE2L2, HMOX1 and TXN in both unstimulated and ROS-stimulated conditions (Fig. 4a–e). In contrast, ectopic expression of wild type SETD7 inhibited the transcription of *NFE2L2*, *SOD2*, and *CAT* (Fig. 4f–i). As NFE2L2 is subject to post translational modifications, which then regulate its stability and activities, we hypothesized that SETD7 may directly interact and inhibit/destabilize NFE2L2 protein. FLAG-tagged SETD7 and NFE2L2 plasmids were transfected into HEK293 cells to enable protein pull-down studies. NFE2L2 protein was detected from immunoprecipitation using anti-FLAG antibody and cell lysate from co-expression of NFE2L2 and FLAG-tagged SETD7 but not from cells transfected with NFE2L2 or SETD7 alone (Fig. 4j,k). The direct interaction between NFE2L2 and SETD7 was also confirmed by *in vitro* pull-down studies, in which FLAG-tagged recombinant NFE2L2 was able to bind to the purified SETD7 protein in cell-free system (Fig. 4l). These results suggest that SETD7 can interact with NFE2L2 and directly regulate its stability and function in response to oxidative stress.

### SETD7 lysine methyltransferase activity is necessary for ROS signaling

**Figure 5:**
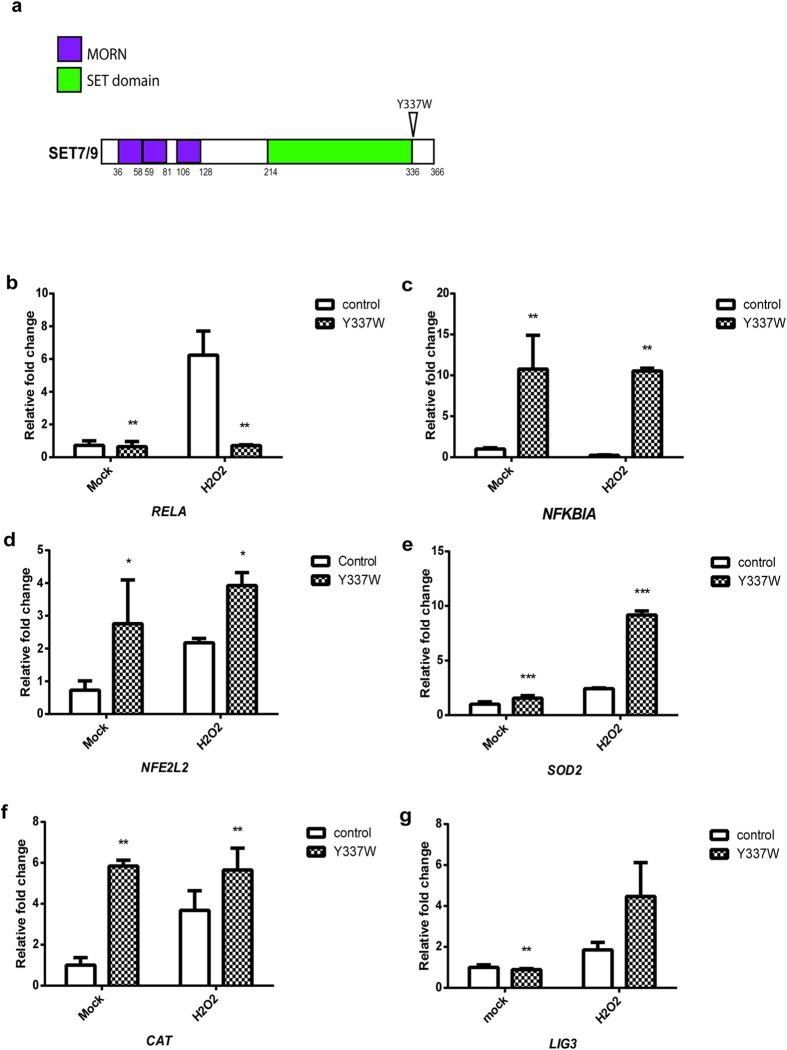
SETD7 methyltransferase activity is required for its regulatory roles on ROS signaling. (**a**) Schematic representation of SETD7 functional domains and the location of Y337W. (**b–g**) Transcription levels of *RELA, NFKBIA, NFE2L2, SOD2, CAT* and *LIG3* were determined by qRT-PCR using total RNAs extracted from Beas-2B cells transfected with control or SETD7 Y337W plasmids.

**Figure 6:**
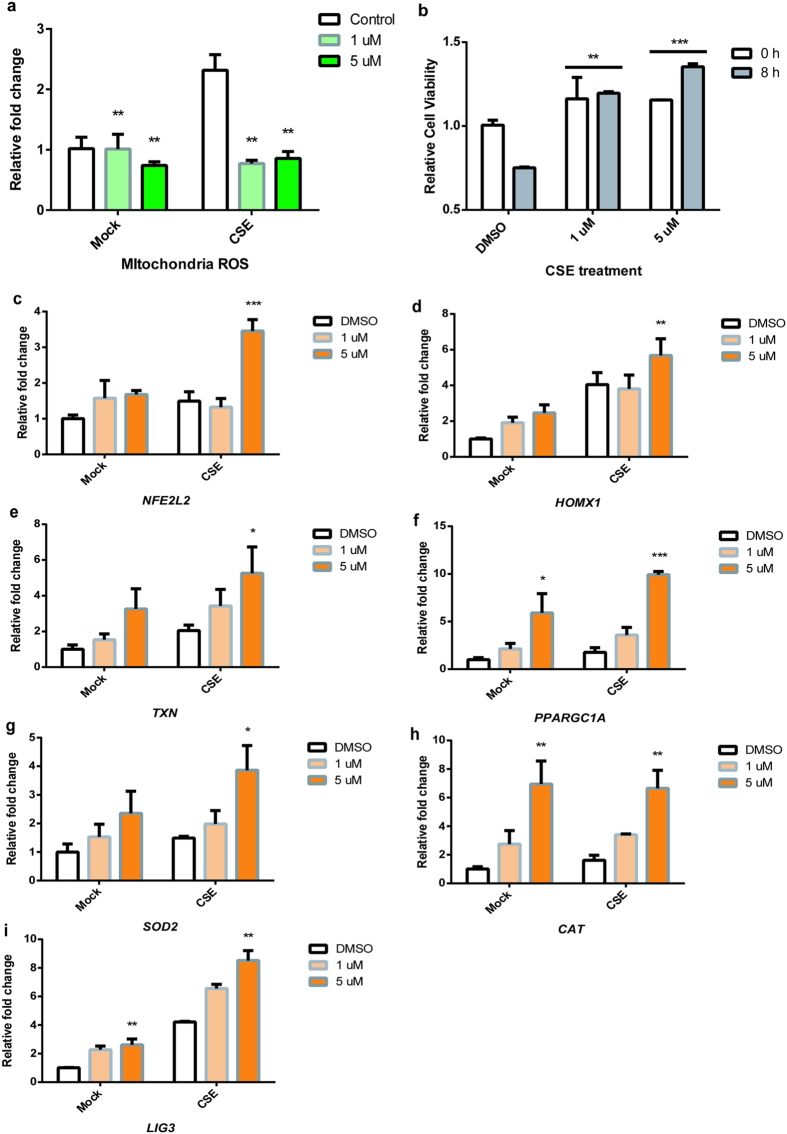
SETD7 inhibitor is able to alleviate ROS accumulation. (**a**) Quantification of CSE-induced mitochondrial ROS production with either DMSO or different doses of (*R*)-PFI-2 in NHLFs by MitoSox. (**b**) NHLFs viability under CSE treatment at the presence of (*R*)-PFI-2 was determined by WST-1 assay at 0- and 8-hour time point. ** indicates *P* < 0.01, *** indicates *P* < 0.001 (Student’s unpaired t test). (**c–i**) Transcription levels of *NFE2L2*, *PPARGC1A*, *HMOX1, TXN, SOD2*, *CAT* and *LIG3* were assessed by qRT-PCR using total RNAs from Beas-2B cells treated with SETD7 inhibitor under CSE-induced oxidative stress. Data were expressed as mean ± standard error of the mean (SEM). n = 3. * indicates *P* < 0.05; ** indicates *P* < 0.01; *** indicates *P* < 0.001 (two-way ANOVA).

Next we tested whether lysine methylation is required for the SETD7-mediated oxidative stress responses. A single amino acid mutation of tyrosine to tryptophan (Y337W) located at the SETD7 substrate binding site was previously identified as a mutation that abrogates SETD7 catalytic activity^46^ (Fig. 5a). By transfection of SETD7 Y337W vectors into Beas-2B cells, we observed a dominant-negative effect of the mutant protein, in which transcription levels of *RELA* were reduced while *NFKBIA*, *NFE2L2*, *SOD2*, *CAT* and *LIG3* mRNA levels were increased (Fig. 5b–g). In order to confirm that under oxidative stress, ROS regulation depends on the catalytic activity of SETD7, we used (*R*)-PFI-2, a highly selective small molecule which can bind to SETD7 and inhibit its methyltransferase activity^46^, to evaluate ROS levels following incubation in cells. Under CSE stimulation, (*R*)-PFI-2 was able to mimic the effects of SETD7 silencing: Incubation with (*R*)-PFI-2 resulted in reduction of mitochondria ROS in Beas-2B cells (Fig. 6a). Inhibition of SETD7 methyltransferase activity by (*R*)-PFI-2 also contributed to improved cell survival rate against oxidative stress induced by CSE (Fig. 6b). Furthermore, *NFE2L2* and PPARGC1A, as well as mitochondrial enzymes *SOD2*, *CAT* and *LIG3* mRNAs were upregulated when cells were incubated with (*R*)-PFI-2 (Fig. 6c–i), suggesting that the lysine methyltransferase activity of SETD7 is necessary to its mitochondria regulatory functions and ROS signaling.

## Discussion

**Figure 7:**
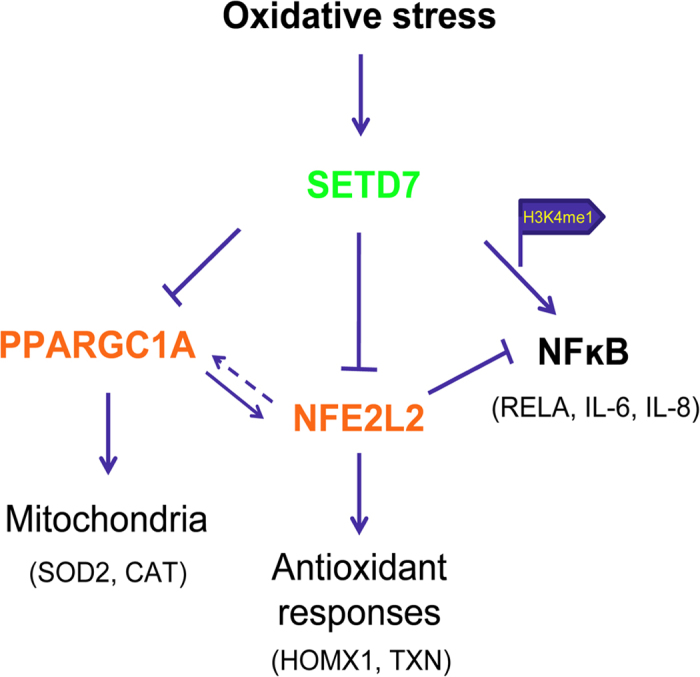
A proposed model for the regulatory roles of SETD7 in the oxidative stress response and ROS signaling. SETD7 mediates redox signal through both transcriptional regulation and post-translational modification: 1) SETD7 modulates mitochondria functions through negative regulation of PPARGC1A activity; 2) SETD7 inhibits the expression of *NFE2L2* and its downstream antioxidant genes (*HOMX1*, *TXN*). This may be due to the interaction between NFE2L2 and SETD7 that leads to methylation and inhibition of NFE2L2 stability. 3) Through histone 3 lysine 4 monomethylation (H3K4me1), SETD7 stimulates the transcription activity of NF-ĸB and its downstream cytokine IL-6 and IL-8 production in response to ROS stimulation.

A central question to oxygen signaling is how ROS homeostasis is regulated. Here we report data generated using *SETD7* RNAi knock-down, overexpression of SETD7 and a pharmacological inhibitor of SETD7, suggesting that lysine methylation by SETD7 regulates the homeostasis of ROS as well as mitochondrial functions. The mechanisms by which SETD7 exerts adverse impact on oxidative stress defense can be explained from the following aspects: 1) Up-regulation of inflammatory cytokine expression (such as IL-6 and IL-8) via transcriptional activation of NF-ĸB; 2) Negative regulation of mitochondria biogenesis via repression of PPARGC1A, LIG3 and ROS detoxifying enzymes such as SOD2 and CAT; 3) Modulation of oxidative stress response by suppressing the expression of *NFE2L2* and its downstream anti-oxidant genes (*HMOX1* and *TXN*) (Fig. 7). Thus, targeting SETD7 may improve cellular resistance against both oxidative injury and energy-deprivation induced damage as well.

Our study demonstrates that the diverse function of SETD7 on oxidative stress is mediated through regulation of three key transcriptional regulators (NF-ĸB, PPARGC1A, and NFE2L2). However, the detailed mechanism of how SETD7 regulates these proteins is presently unclear. Several hypotheses are described below.

### Regulation of NF-ĸB

The classical function of SET domain methyltransferases is modulation of core histones. The reduced H3K4me1 level in the *RELA*/p65 promoter in SETD7 knockdown cells indicates that SETD7 promotes NF-ĸB and pro-inflammatory cytokine production in response to ROS stimulation at least in part mediated through H3K4me1 transcriptional activation. Notably, our observation is in consistent with the previously reported hyperglycemia model in which SETD7 drives *RELA* regulation *via* histone modification^17^,^47^. The resulting inflammatory response mediated by SETD7-induced NF-kB activation and cytokine production is likely to exacerbate tissue damage from the oxidative stress in various disease conditions.

### Regulation of PPARGC1A

Mitochondria are fundamental to metabolism and cellular signaling. PPARGC1A is the pivotal factor for these functions and can be negatively regulated by SETD7. Originally considered as a thermogenic coactivator, PPARGC1A orchestrates a variety of mitochondrial functions, including mitochondria biogenesis and mitochondrial ROS clearance, and has been identified as a crucial player in metabolic regulation^25^,^26^,^48^,^49^. Our results showed that knockdown or inhibition of SETD7 may improve resistance to ROS damages through mitochondrial biogenesis and production of mitochondrial detoxifying enzymes, both of which can be achieved by PPARGC1A up-regulation. The mechanism by which SETD7 coordinates PPARGC1A activity is presently unclear. As SETD7 is known to catalyze the H3K4me1 and activate transcription, the positive effect of H3K4me1 on transcriptional activation would not readily explain the suppressive role of SETD7 on PPARGC1A. One of the possible scenarios is that SETD7 mediates PPARGC1A activities via methylation of another protein. SIRT1 has been identified as a direct regulator of PPARGC1A and a non-histone substrate of SETD7^11^. It is tempting to speculate that SETD7 may methylate SIRT1 and thus preventing its activation effect on PPARGC1A. Determining whether SETD7 can influence PPARGC1A through SIRT1 would provide new insight into the regulatory mechanisms of mitochondrial signaling.

### Regulation of NFE2L2

NFE2L2 nuclear transcription factor is a key regulator of the cellular defense mechanism that combats oxidative stress by inducing activation of detoxifying and antioxidant genes. NFE2L2 is known to be regulated mainly by KEAP1^32^,^33^. Additionally, post translation modification of NFE2L2 such as ubiquitination, phosphorylation and acetylation was also found to participate in the regulation of its stability/activity dependent or independent of KEAP1 interaction^32^,^33^. In this study we showed that SETD7 can bind to NFE2L2 protein in both an overexpressing system and a cell-free system using purified proteins (Fig. 4k, l) SETD7 negatively regulates the expression of NFE2L2 and its downstream genes. Furthermore, inhibition of the catalytic activity of SETD7 by either mutant SETD7 or SETD7 selective small molecule inhibitor resulted in increased NFE2L2. It is possible that SETD7 may directly methylate one or more lysine residues in NFE2L2. The methylation then reduces the overall NFE2L2 protein levels by facilitating KEAP1-NFE2L2 interaction and accelerating its degradation. The increased NFE2L2 protein in the SETD7-knockdown or compound-treated cells may provide a positive feedback mechanism for its own transcription by binding to the ARE region in the NFE2L2 promoter^50^, leading to the concurrent increase in the *NFE2L2* mRNA levels (Figs 4a and 5c). Further studies will be required to determine the possibility of direct methylation of NFE2L2 by SETD7 and whether the methylation status of NFE2L2 is altered by ROS.

### Cross talk

Early studies on the mechanisms of mitochondrial biogenesis showed that ectopic expression of PPARGC1A strongly induced the expression of NFE2L2^24^. Therefore, our findings on the upregulation of NFE2L2 by inhibition of SETD7 may be a combination of direct regulation of NFE2L2 and indirectly via PPARGC1A. Reciprocally, as a transcription factor, NEF2L2 is able to drive the induction of PPARGC1A^51^. Furthermore, NFE2L2-driven antioxidant effects also contribute to the attenuation of NF-ĸB^30^,^52^. Thus, the cross talk among these pathways can also contribute to the final outcome of SETD7 inhibition.

The SETD7-mediated ROS regulation reflects both the transient, dynamic characteristic of epigenetic changes and the potential existence of post-translational modification, both of which are necessary to rapidly trigger multifaceted responses in accordance to extracellular environment. Since SETD7 knockout mice are phenotypically normal^18^,^19^, these findings provide new clues to interpret the roles of SETD7 *in vivo*. We speculate that the SETD7 knockout may afford a protective effect in the oxidative stress response. Further evaluation of SETD7 knockout mice under the oxidative stress condition is warranted to confirm the hypothesis.

Taken together, we report for the first time that SETD7 coordinates oxygen signaling *via* activation of NF-ĸB and suppression of metabolic master regulator PPARGC1A and NFE2L2/ARE pathway. Our data suggest that SETD7 may regulate ROS signaling not only through transcription, but also likely through interaction and modification of NFE2L2. In addition, we provide data to reveal the potential benefit of a SETD7 small molecule inhibitor in combating oxidative stress primarily by up-regulation of PPARGC1A and NFE2L2 genes. To gain complete understanding of the molecular mechanisms regulated by SETD7 in these pathways, we are currently investigating transcriptional regulation directly through SETD7 as well as direct interaction and methylation of proteins responsible for ROS signaling and mitochondrial functions. In addition, further studies in mouse models to test the effect of SETD7 inhibition would be necessary to evaluate the therapeutic potential of targeting SETD7 in treating various ROS-associated diseases including inflammation, neurodegeneration and metabolic disorders.

## Materials and Methods

### Cell Culture and treatment

Human lung bronchus epithelial cells (Beas-2B) and human kidney epithelial cells (HEK293) were obtained from ATCC and maintained following standard procedures. Normal human lung fibroblasts (NHLF) were purchased from LONZA and cultured following manufacturer’s instruction. Briefly, BEGM bronchial epithelial cell growth medium with BEGM bulletkit (LONZA) was used for the growth and maintenance of Beas-2B cells. NHLFs were cultured in Fibroblast Cell Medium BulletKit (LONZA). Cells were maintained at 37 °C, 5% CO_2_ and split every 3–4 days when the cells reached ~80% confluence. HEK293 cells were cultured in ATCC-formulated Eagle’s Minimum Essential Medium supplemented with 10% fetal bovine serum. Human monocytes were isolated from leukopak (Massachusetts General Hospital) using RosetteSep Human Monocyte Enrichment Cocktail (StemCell Technologies). The enriched monocytes were then treated with GM-CSF to derive into macrophages. Cells were then starved for 24 hours in serum-free medium before any treatment. Cigarette smoke extract (CSE) was prepared using the method described before^41^. To induce oxidative stress, 0.1 AU/ml CSE or 0.1 μM hydrogen peroxide (H_2_O_2_) was added to the cultured media and incubate for different time length at 37 °C. Mock control without stimulation was included in each individual experiment. Compound tests were performed using similar procedure. DMSO was used as controls in all the compound experiments.

### siRNA knockdown

ON-TARGETplus SMARTpool of human SETD7 siRNAs and scramble siRNAs were obtained from Dharmacon. siRNA transfection was performed using the Oligofectamine reagent (Life Technologies) in 96-well plate format. 48 hours after transfection, cells were washed and recalibrated before stimulated by CSE or H_2_O_2_. GM-MDM derived macrophage siRNA transfection was performed using Nucleofector kit for human macrophages (LONZA) on Amaxa nucleofector device (LONZA) following manufacturer’s protocol.

### Plasmids and transfection

DDK (FLAG)-tagged SETD7 human ORF clone, NFE2L2 human cDNA clone and pCMV6-DDK tagged empty vectors were obtained from Origene. SETD7 Y337W mutant construct was generated by subcloning the SETD7 cDNA carrying Y337W mutation into the pcDNA 3.1/Myc-His plasmid, followed by sequencing to confirm cloning accuracy. Plasmids were prepared by EndoFree Plasmid Maxi kit (QIAGEN). Plasmids were delivered to the cells using Lipofectamine LTX with Plus Reagent (Life Technologies). Briefly, cells were plated in 6-well plates. The transfected cells were incubated at 37 °C for 48 hours before treatment. pCMV6-DDK tagged vectors were transfected in the same manner and serve as controls.

### Cytokine quantification by ELISA

The MSD human Proinflammatory-4 II Ultra-Sensitive kit (Meso Scale Discovery) was used to quantify the production of IL-6 and IL-8 following manufacturer’s instruction. Each sample was tested in triplicates in every experiment. MSD plates were analyzed by MS2400 imager from MSD. Duplicate of standards were included in each plate to generate standard curve for data interpretation.

### Western Blot

Immunoblotting was performed to detect protein expression using the Quick Western Kit–IRDye® 680RD (LI-COR Biosciences) following standard protocol. Primary antibodies used were: anti-SETD7 (a gift from Dr. Susanne Gräslund at SGC), anti-H3K4me1, anti-Histone H3, anti-NFE2L2, Anti-PPARGC1A (Abcam), anti-DDK (Origene), anti-SOD2 (Abcam). Membrane transfer was performed on iBlot 2 Gel Transfer Device with iBlot 2 Transfer stacks (PVDF) (Life Technologies). Imaging analysis was performed on the Odyssey CLx Infrared Imaging System using Image Studio Ver 3.1 (LI-COR Biosciences).

### Pull-down assay

Protein pull-down was performed with Anti-DDK Magnetic Immunoprecipitation Kit (Origene). Beads were preblocked at 4 °C before use. Transfected cell lysates were precleared with control magnetic beads for at least 2 hours at room temperature. Supernatant was saved for the immuneprecipitation procedure. Each immunoprecipiation mix was incubated overnight at 4 °C followed by at least 3-time wash before the elution step. For *in vitro* pull-down assay, FLAG-tagged human NFE2L2 recombinant protein (Origene) was incubated with Anti-DDK magnetic beads (Origene) for 1 hour at room temperature. GST-tagged human SETD7 recombinant protein (a gift from Dr. Masoud Vedadi at University of Toronto) was added to the beads and incubated for another hour at room temperature. After 4-time wash with wash buffer, the IP was eluted by 2X SDS-sample buffer. Samples were stored at - 80 °C or directly used for Western blot analysis.

### Chromatin immuneprecipitation

Chromatin from Beas-2B cells treated by scramble or siSETD7 siRNA with or without H_2_O_2_ stimulation were fixed with 37% formaldehyde. Chromatin immunoprecipitation was performed with anti-H3K4me1 (Active Motif) and ChIP-IT Express Chromatin Immunoprecipiation Kit from Active Motif, following protocols from the manufacturer. 20 ug chromatin from each treatment group and 5 μl of anti-H3K4me1 or anti-IgG antibody were used for each IP. For qPCR, primer pairs corresponding to the −250 bp ~+250 bp *RELA* promoter were designed. GAPDH (positive control) primer pairs and two primer pairs (negative controls) that amplify regions in gene deserts (Untr4, Untr12) were included in each test. Relative expression level of each site was shown by comparison to input chromatin of each sample.

### RNA isolation and qPCR analysis

Total RNA from cell pallets was isolated by RNeasy mini kit (QIAGEN) and treated with RNase-Free DNAse set (QIAGEN) to remove genomic DNA. Each RNA sample was measured by Nanodrop to determine the quantity and quality. TaqMan probes are designed and generated by Life Technologies. Sequences are available on request. qPCR was performed using the ViiA 7 Real-Time PCR System (Life Technologies) with the Taqman RNA-to-Ct 1-step kit (Life Technologies). The threshold (CT) was set at a point where the fluorescence signal was above the baseline noise but as low as possible in the exponential amplification phase. Experiments were performed in triplicates. Relative expression of genes was compared to GAPDH in each individual experiment.

### Cytotoxicity measurement

Formazan-based cytotoxicity assay was used to measure cytotoxicity of cells in 96-well format in triplicates. Each experiment was repeated at least 3-times independently. The amount of formazan dye formed by metabolically active cells was measured by SpectraMax M4 Multi-Mode Microplate Reader (Molecular Devices). Relative cell viability was represented by comparing the absorbance of sample wells to blank well reads at each time point.

### Quantification of intercellular ROS and mitochondrial superoxide

To quantify intracellular ROS, OxiSelect™ Intracellular ROS Assay Kit (Cell Biolabs) was used following the manufacturer’s instruction. Fluorescence intensity was measured with spectrofluorometer plate reader. Standard curve was included in every independent assay. MitoSOX™ Red mitochondrial superoxide indicator (Life Technologies) was applied to determine mitochondrial superoxide level following manufacturer’s instruction. Mitochondria ROS quantification in 96-well format was performed using protocols described elsewhere^22^. Briefly, MitoSOX reagent in working solution was added to each well after stimulation and incubated at 37 °C in the dark. Lysis buffer was added after three time wash before measurement. The measured fluorescence value was expressed as a fold change compared with that of untreated control. For live cell imaging, MitoSox reagent working solution was loaded to cover cells and incubated at 37 °C in the dark. Cells were then washed gently before imaging acquisition. Nuclei were counterstained with DAPI. Images were captured and processed with Nikon ECLIPSE Ti Inverted Research Microscope and NIH Image J.

### Mitochondria assessment

Visualization of mitochondria within cells was performed with MitoTracker Green (Invitrogen). Live cells were grown on coverslips and treated as described above. After stimulation, cells were washed with warm buffer and incubated with MitoTracker Green at 37 °C. Cells were gently washed three times. VECTASHIELD^®^ Mounting Media (Vector Laboratories) was added to each slide before image capture. At least three slides were prepared from each treatment group, and three individual areas were examined per slides for mitochondria labeling.

### Statistical analysis

Each experiment was repeated at least three times in triplicates. Data of each figure consist of a representative experiment of the independent set of experiments. Statistical analyses were performed using Prism 6 (GraphPad Software). Relative fold change of mRNA level was quantified by comparison with cells transfected with scramble siRNA without stimulation (mock), unless specified in the figure legends. Data were expressed as mean ± standard error of the mean (SEM). Statistical significance was determined by comparing the means between groups by Student’s t test or two-way ANOVA. *p* < 0.05 (*) was considered statistically significant.

## Additional Information

**How to cite this article**: He, S. *et al.* Lysine Methyltransferase SETD7 (SET7/9) Regulates ROS Signaling through mitochondria and NFE2L2/ARE pathway. *Sci. Rep.* **5**, 14368; doi: 10.1038/srep14368 (2015).

## Supplementary Material

Supplementary Information
