# Supplementary material for: Lysine Methyltransferase SETD7 (SET7/9) Regulates ROS Signaling through mitochondria and NFE2L2/ARE pathway
Source: Sci Rep. 2015 Oct 5;5:14368. doi: 10.1038/srep14368 (PMC4593030; doi:10.1038/srep14368)
Supplement: Supplementary Information [file srep14368-s1.pdf]

Lysine Methyltransferase SETD7 (SET7/9) Regulates ROS Signaling through mitochondria and NFE2L2/ARE pathway

Shuying He[a], Dafydd R. Owen[b], Scott A. Jelinsky[a] and Lih-Ling Lin[a]*

[a]Inflammation and Immunology Research Unit, Pfizer Research

[b]Worldwide Medicinal Chemistry, Pfizer Worldwide Research and Development, Cambridge, MA 02139

Additional Information

Supplementary Figure S1. SETD7 regulates NF-κB and inflammatory responses in human macrophages under oxidative stress.

Supplementary Figure S2. Correlation of SETD7 and NFE2L2 (NRF2) expression in sputum from COPD patients.

Supplementary Figure S3. Relative expression levels of genes in scramble and SETD7 siRNA-transfected cells with CSE or H2O2 treatment compared to that in scramble siRNA-transfected cells without treatment with CSE or H2O2.

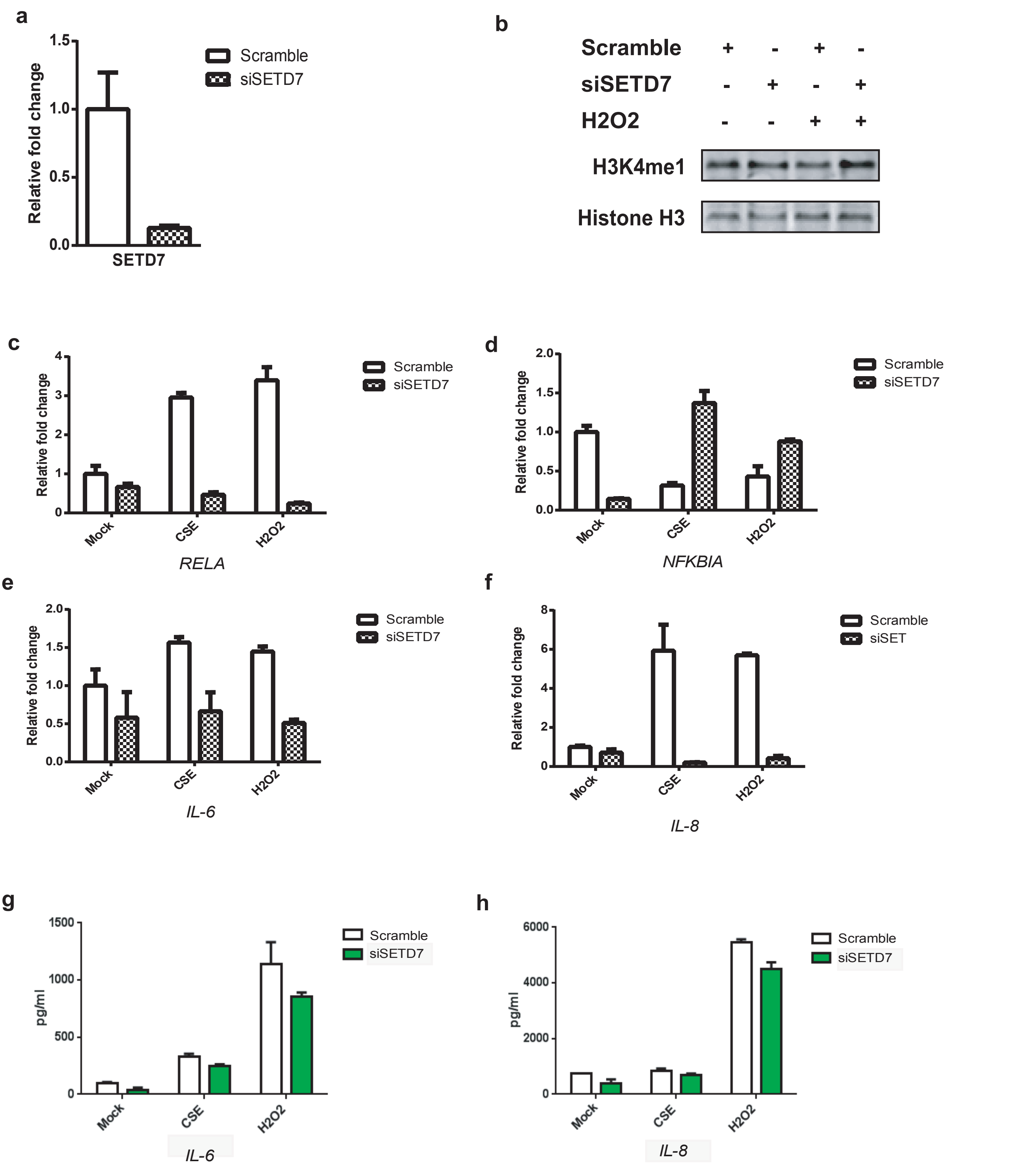

Supplementary Figure S1. SETD7 regulates NF-κB and inflammatory responses in human macrophages under oxidative stress.

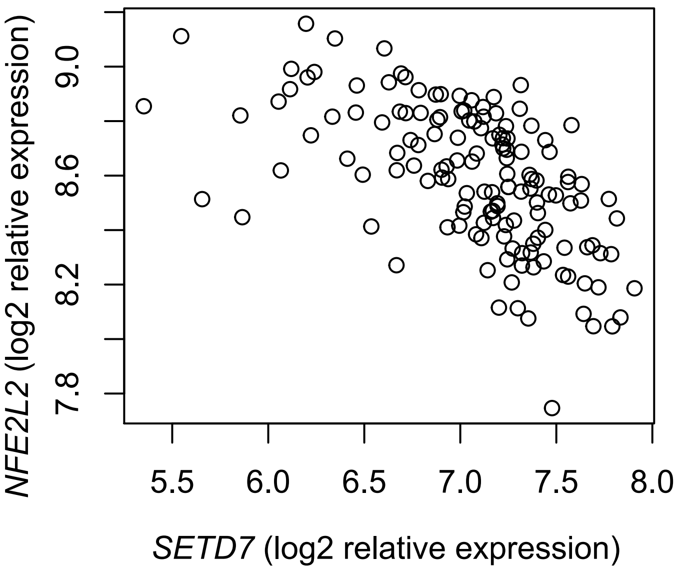

Supplementary Figure S2. Correlation of expression of SETD7 and NFE2L2 in sputum COPD patients. Expression data was obtained from the Evaluation of COPD Longitudinally to Identify Predictive Surrogate Endpoints (ECLIPSE)1 study of ex-smoker COPD subjects. Data was downloaded from the Gene Expression Omnibus (GEO) database (http://www.ncbi.nlm.nih.gov/geo/query/acc.cgi?acc=GSE22148). The expression of SETD7 and NFE2L2 has a correlation of -0.5853.

Reference:
Singh, D. et al. Induced sputum genes associated with spirometric and radiological disease severity in COPD ex-smokers. Thorax 66, 489-495, doi:10.1136/thx.2010.153767 (2011).

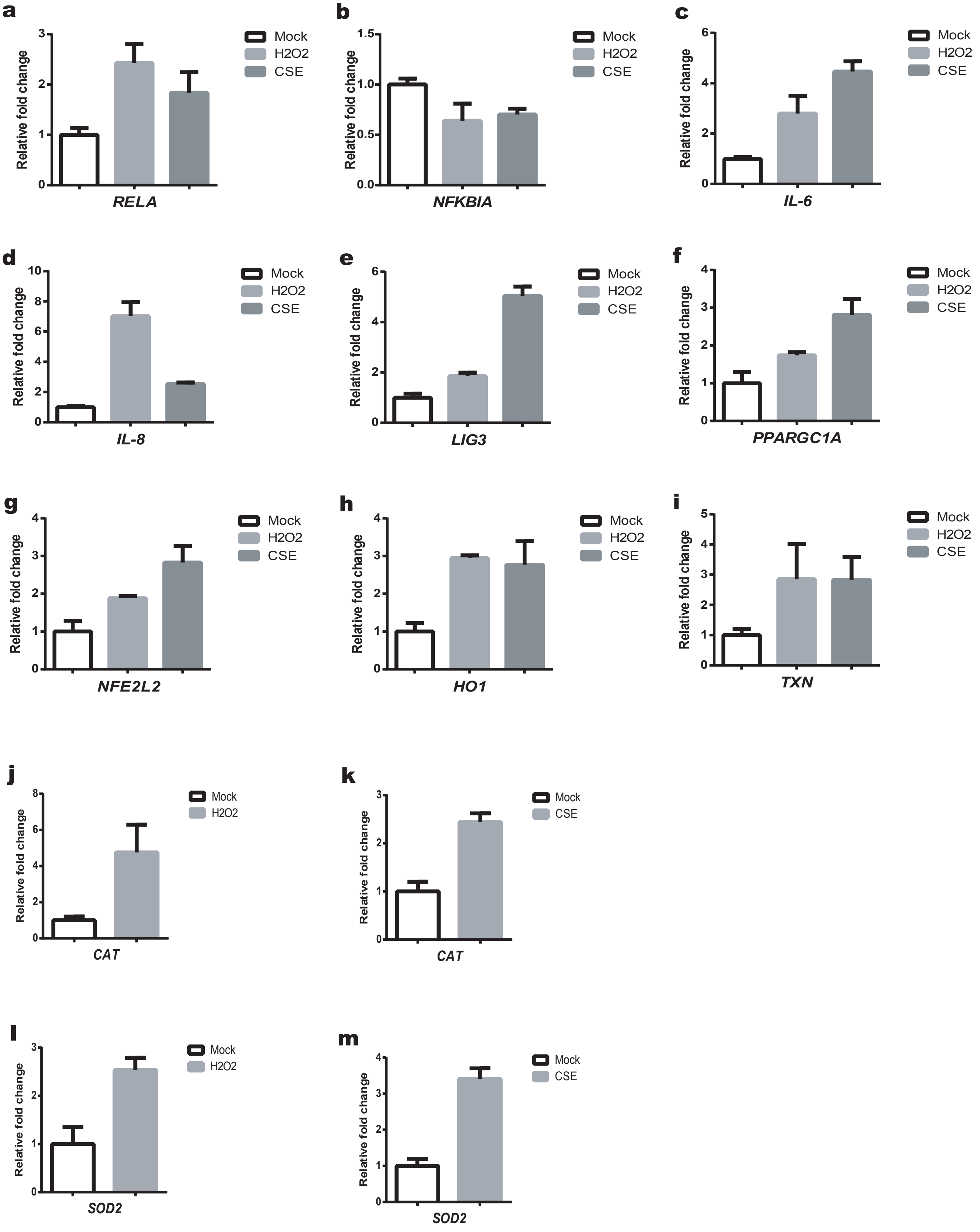

Supplementary Figure S3. Relative expression levels of genes in scramble and SETD7 siRNA -transfected Beas-2B cells with CSE or H2O2 treatmend compared to that in scramble siRNA -transfected cells without treatment.
